# Supplementary material for: Recurrent Phases of Strict Protein Limitation Inhibit Tumor Growth and Restore Lifespan in A Drosophila Intestinal Cancer Model
Source: Aging Dis. 2024 Feb 1;15(1):226–44. doi: 10.14336/AD.2023.0517 (PMC10796089; doi:10.14336/AD.2023.0517)
Supplement: Supplementary file 1 [file AD-15-1-226-s.pdf]

SUPPLEMENTARY DATA

Recurrent Phases of Strict Protein Limitation Inhibit Tumor Growth and Restore Lifespan in A *Drosophila* Intestinal Cancer Model

Roxana M. Pfefferkorn, Benedikt M. Mortzfeld, Christine Fink, Jakob von Frieling, Judith Bossen, Daniela Esser, Christoph Kaleta, Philip Rosenstiel, Holger Heine, Thomas Roeder

© 2023. Pfefferkorn RM et al. Published online at http://www.aginganddisease.org/EN/10.14336/AD.2023.0517

SUPPLEMENTARY DATA

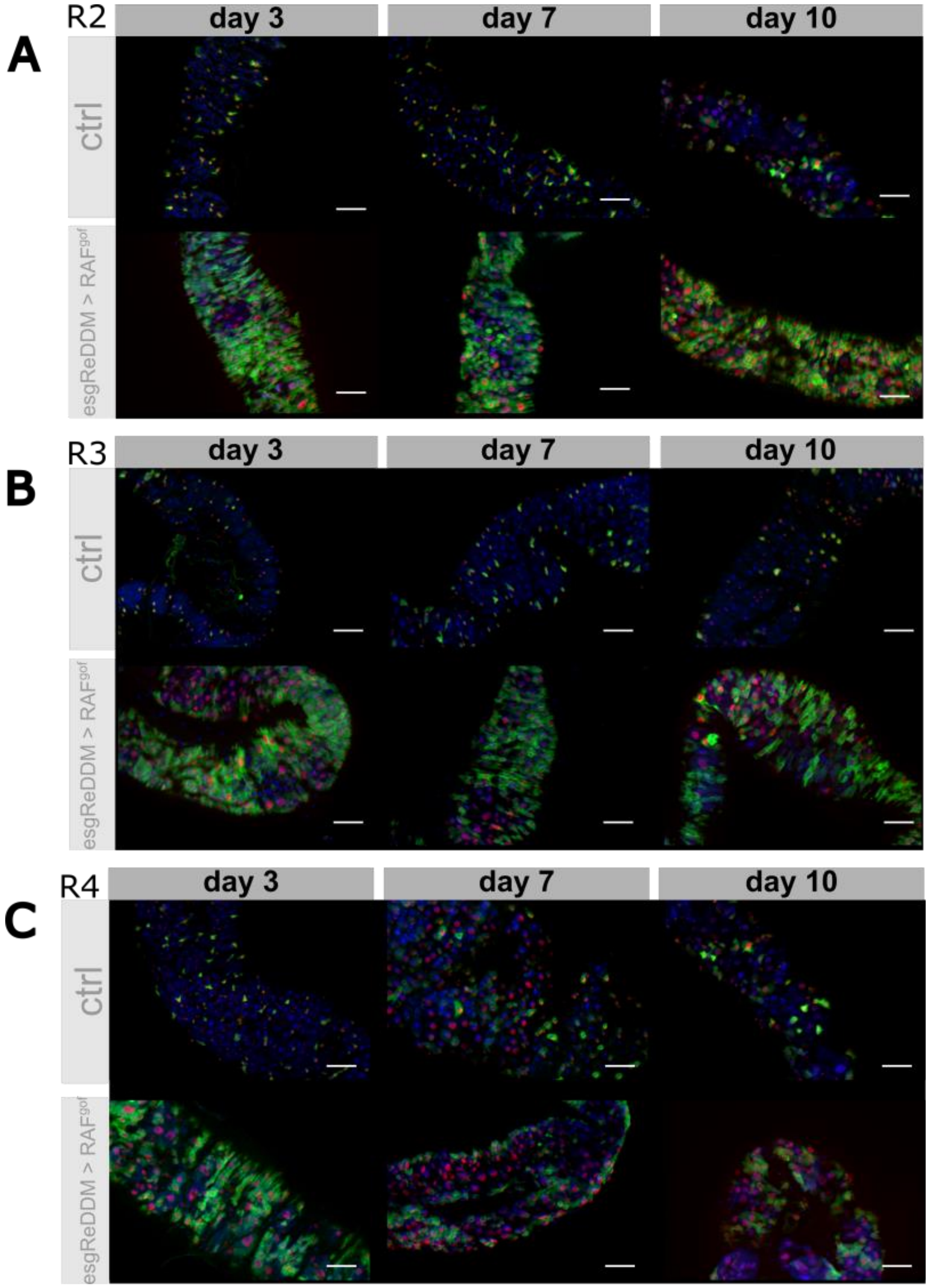

Supplementary Figure 1. Lineage tracing in midgut region R2 (A), R3 (B), and R4 (C) of control flies and flies expressing RAF^{gof} in ISCs and EBs. The ReDDM system (1) was used to visualize the development of ISCs (expressing GFP) into enteroblasts (expressing RFP). Intestines were dissected after 3, 7 or 10 days of tumor induction from flies on control diet. During differentiation the short-lived GFP fades, leaving fully differentiated enteroblasts marked with RFP only (scale bar 50µm). ctrl = esgReDDM > w1118.

(1) Antonello ZA, Reiff T, Ballesta-Illan E, Dominguez M. Robust intestinal homeostasis relies on cellular plasticity in enteroblasts mediated by miR-8-Escargot switch. EMBO J. 201534(15):2025-2041.

© 2023. Pfefferkorn RM et al. Published online at http://www.aginganddisease.org/EN/10.14336/AD.2023.0517

SUPPLEMENTARY DATA

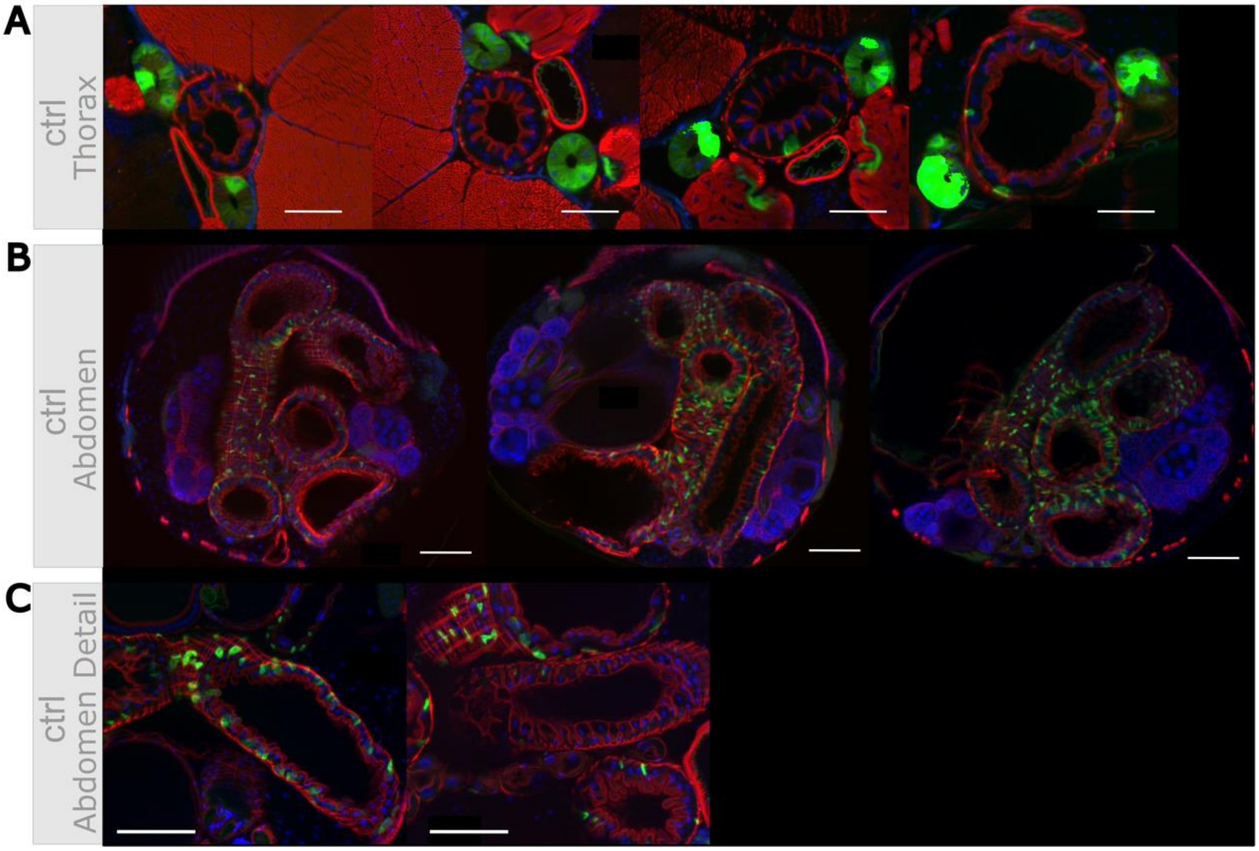

Supplementary Figure 2. Intestinal structure and phenotype variability of healthy control flies and oncogene expressing flies on a nutritious control diet. A-C, top: control flies (EGT;Luc2 > w^{1118}) and A-C, bottom: oncogene-bearing flies (EGT;Luc2 > RAF^{gof}) after 3 days of induction, showing (A) sections through the thorax (anterior midgut, R2) (scale bar 50 µm); (B) section showing multiple intestinal loops (R3-5 midgut region) (scale bar 100 µm); and (C) detail of an intestinal loop (region not specified, scale bar 100 µm).

© 2023. Pfefferkorn RM et al. Published online at http://www.aginganddisease.org/EN/10.14336/AD.2023.0517

SUPPLEMENTARY DATA

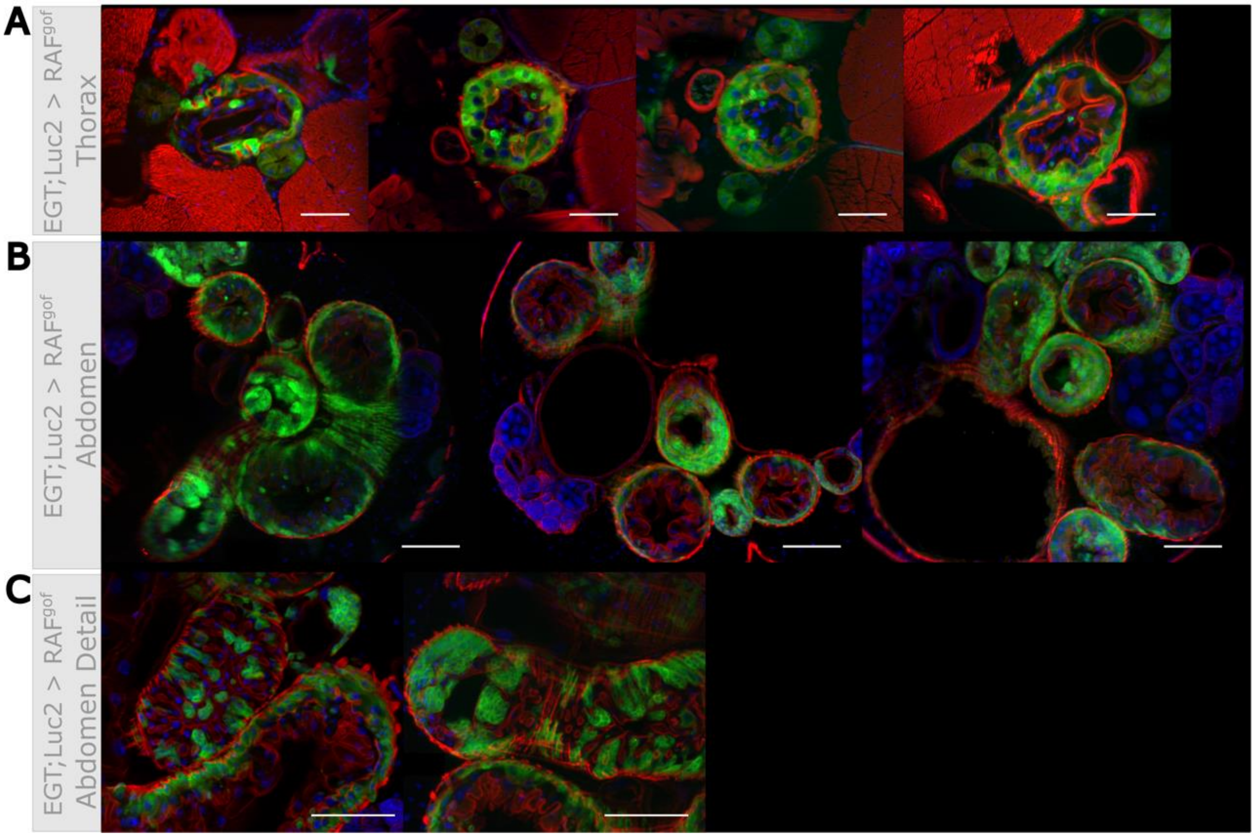

Supplementary Figure 3. Intestinal structure and phenotype variability of healthy control flies (EGT;Luc2 > w^{1118}) on a nutritious control diet. (A) Sections through the thorax (anterior midgut, R2) (scale bar 50 µm). (B) Section through the abdomen (R3-5 midgut region) showing multiple intestinal loops (scale bar 100 µm). (C) Detail of intestinal loop (scale bar 100 µm). The flies were fed for three days on a control diet containing 2% yeast extract.”

© 2023. Pfefferkorn RM et al. Published online at http://www.aginganddisease.org/EN/10.14336/AD.2023.0517

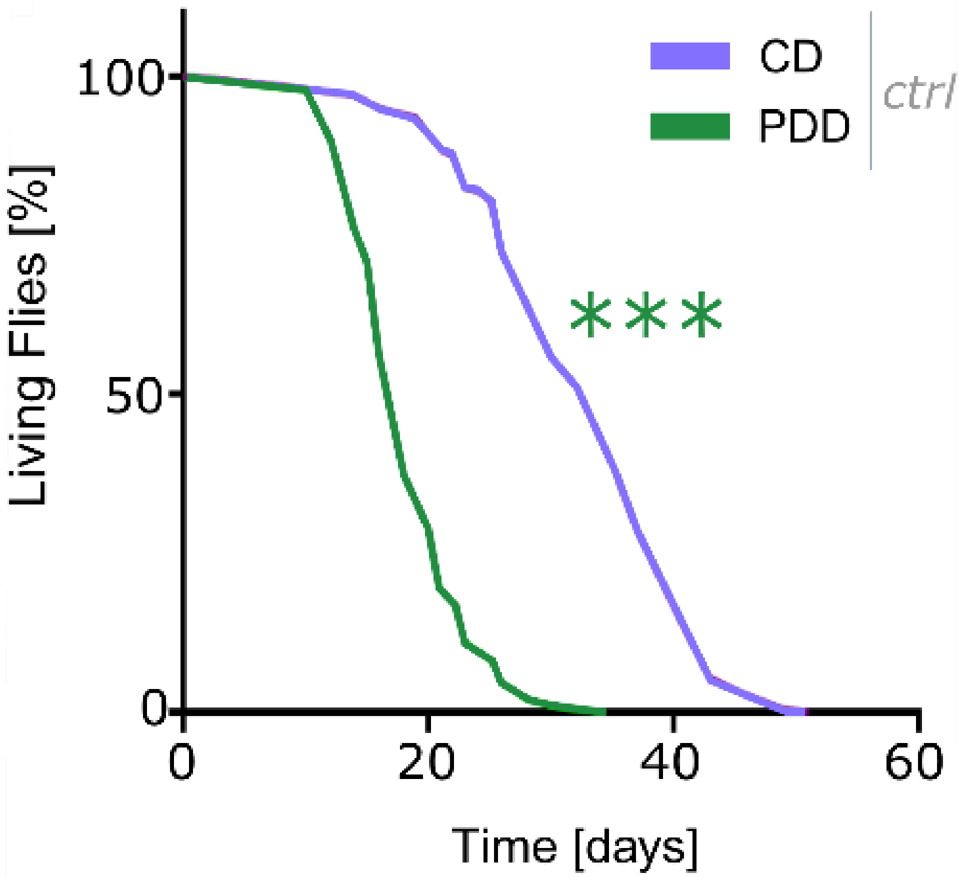

Supplementary Figure 4. Lifespan of control flies subjected to PDD or CD at 29°C (n = 200-205). ctrl = w^{1118}, CD = normal medium, PDD = protein depletion medium, *** $p < 0.001$.

© 2023. Pfefferkorn RM et al. Published online at http://www.aginganddisease.org/EN/10.14336/AD.2023.0517

SUPPLEMENTARY DATA

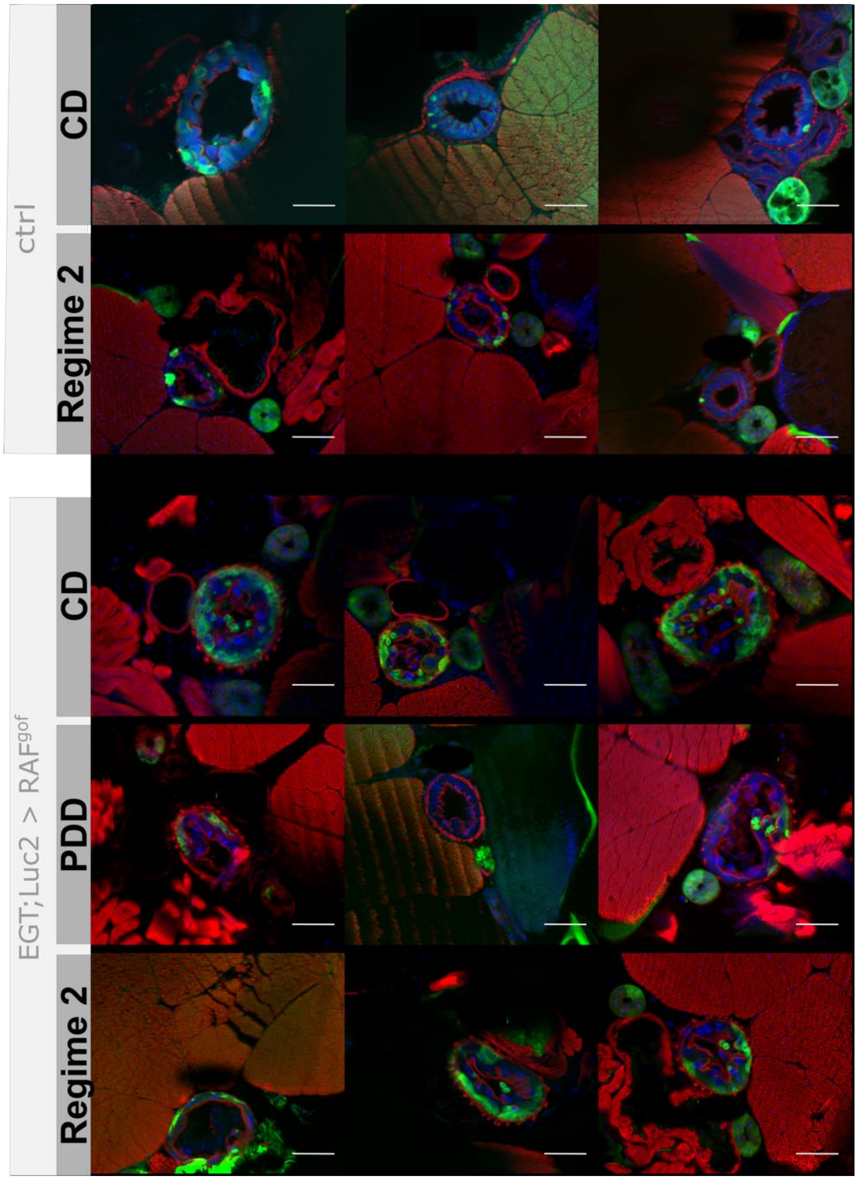

Supplementary Figure 5. Phenotypic observations in cross sections of the anterior R2 midgut region of flies feeding for 10 days on CD, feeding for 3 days on PDD followed by 3 days on CD and 4 days on PDD (PDD/CD) and 10 days on PDD (scale bar 50 µm). ctrl = EGT;Luc2 > w^{1118}, CD = control diet with nutritious medium, PDD = protein depleted diet.

© 2023. Pfefferkorn RM et al. Published online at http://www.aginganddisease.org/EN/10.14336/AD.2023.0517

SUPPLEMENTARY DATA

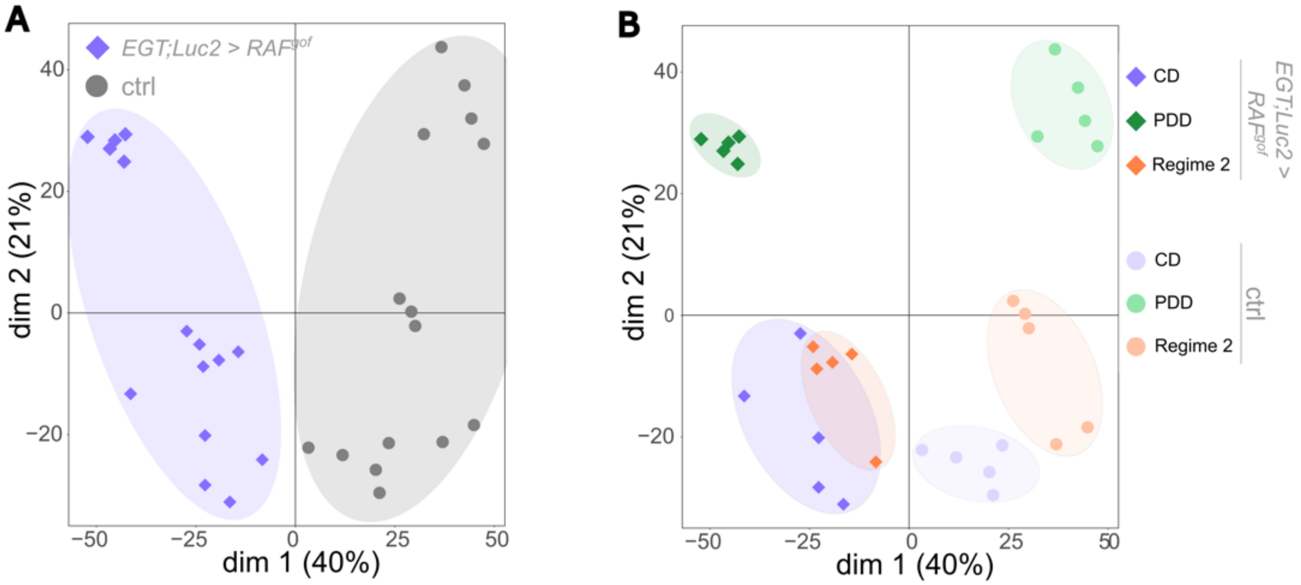

Supplementary Figure 6. PCoA analysis of transcriptomic data. Analysis of variation between and among samples using multi-dimensional scaling (MDS) was based on all transcripts (unweighted). The mRNA of oncogene-bearing and control flies was sequenced after 13 days of subjection to the respective feeding regime (n = 5). (A) Manually added ellipses represent tumor bearing (blue) and healthy controls (grey). (B) The same dataset as in A, now with ellipses representing feeding regimes as well as tumor or control phenotype (n = 5).

© 2023. Pfefferkorn RM et al. Published online at http://www.aginganddisease.org/EN/10.14336/AD.2023.0517

SUPPLEMENTARY DATA

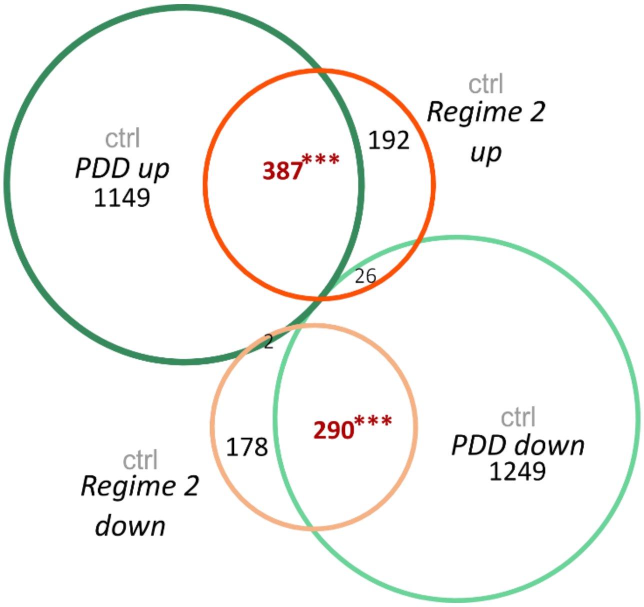

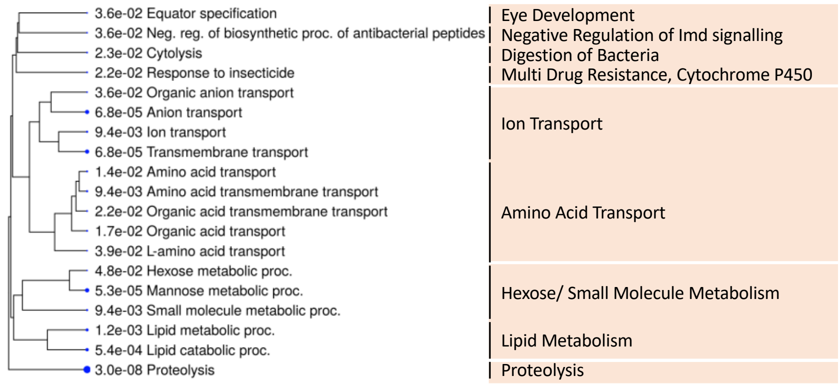

GO analysis of the cohort of genes upregulated in both Regime 2 and PDD

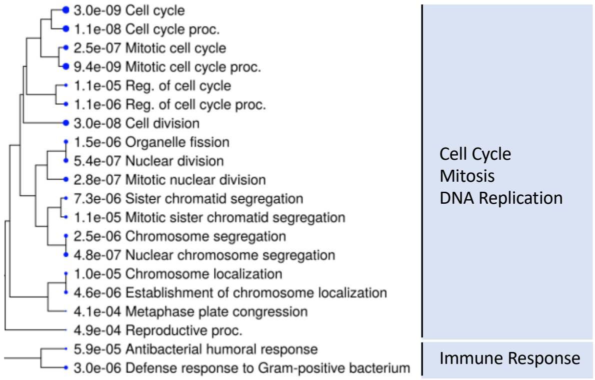

GO analysis of the cohort of genes downregulated in both Regime 2 and PDD

Supplementary Figure 7. Venn diagram identifying genes displaying a transcriptional memory effect (displayed in red). The GO terms of the commonly upregulated genes in Regime 2 and PDD (middle) and of the commonly downregulated genes (bottom) are shown as hierarchical clustering trees. The size of the solid circle corresponds to the enrichment FDR.

© 2023. Pfefferkorn RM et al. Published online at http://www.aginganddisease.org/EN/10.14336/AD.2023.0517

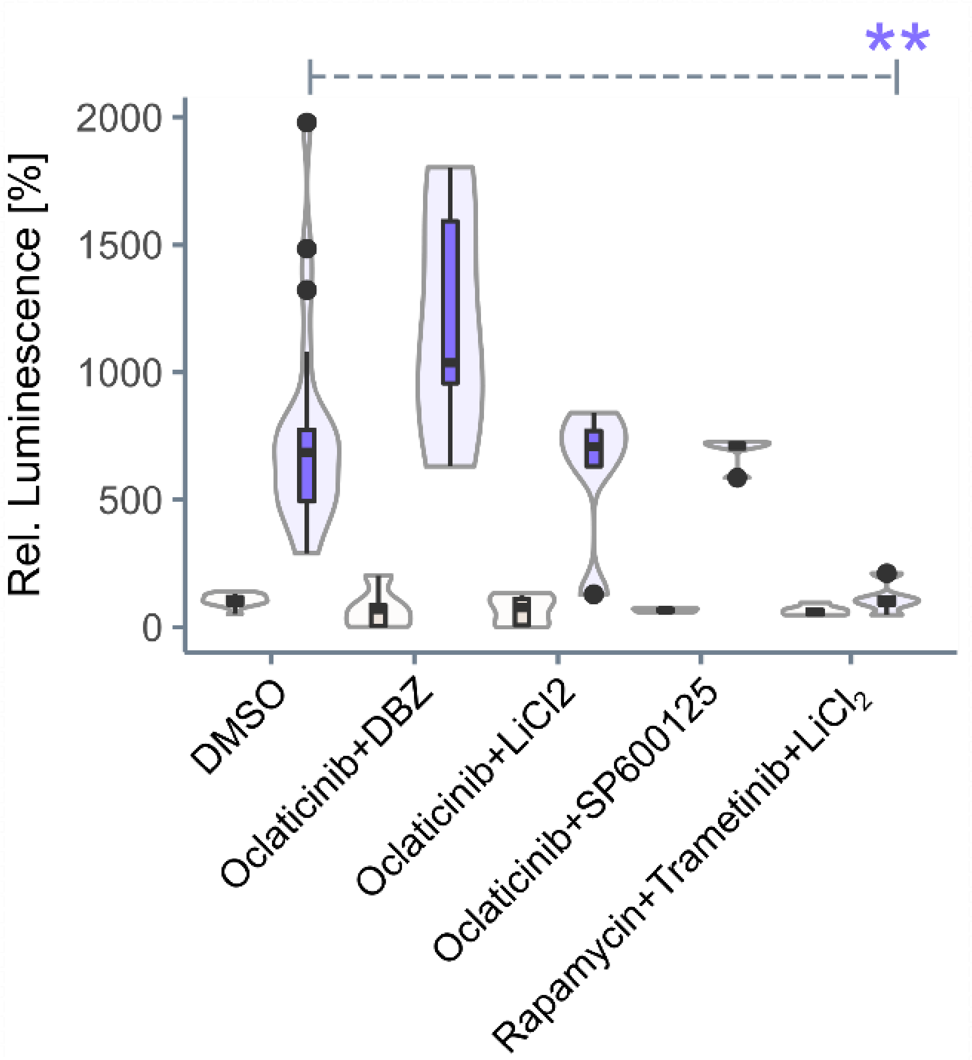

Supplementary Figure 8. Luciferase signal was determined in control flies and in flies expressing RAF^{gof} after 3 days of tumor induction. Flies were fed with CD containing the indicated combinations of pathway-specific inhibitors. Luminescence relative to control flies given DMSO solvent (set as 100%). The combination of rapamycin, trametinib and LiCl2 served as a positive control.

© 2023. Pfefferkorn RM et al. Published online at http://www.aginganddisease.org/EN/10.14336/AD.2023.0517

SUPPLEMENTARY DATA

Supplementary Table 1. Enrichment of GO terms for genes that were downregulated in healthy flies on PDD while upregulated in tumor-bearing flies on CD after 3 days of oncogene induction (n = 5).

Term	Identifier	$log10^{-x}$
cell division	GO:0051301	12.89
mitotic cell cycle	GO:0000278	9.86
spindle organization	GO:0007051	4.18
DNA replication initiation	GO:0006270	3.70
multicellular organismal reproductive process	GO:0048609	3.59
cytoskeleton organization	GO:0007010	3.51
cell differentiation	GO:0030154	1.94
defense response to Gram-positive bacterium	GO:0050830	1.76
anatomical structure development	GO:0048856	1.71
embryonic organ development	GO:0048568	1.69
actin filament -based process	GO:0030029	1.41
animal organ development	GO:0048513	1.34
DNA replication	KEGG:03030	3.42
pentose and glucuronate interconversions	KEGG:00040	3.16
ECM -receptor interaction	KEGG:04512	1.81
retinol metabolism	KEGG:00830	1.63
ascorbate and aldarate metabolism	KEGG:00053	1.49

© 2023. Pfefferkorn RM et al. Published online at http://www.aginganddisease.org/EN/10.14336/AD.2023.0517
